# The Good, the Bad and the Unknown Aspects of Ghrelin in Stress Coping and Stress-Related Psychiatric Disorders

**DOI:** 10.3389/fnsyn.2020.594484

**Published:** 2020-10-27

**Authors:** Eva Maria Fritz, Nicolas Singewald, Dimitri De Bundel

**Affiliations:** ^1^Department of Pharmacology and Toxicology, Institute of Pharmacy and CMBI, University of Innsbruck, Innsbruck, Austria; ^2^Department of Pharmaceutical Sciences, Research Group Experimental Pharmacology, Center for Neurosciences (C4N), Vrije Universiteit Brussel, Brussels, Belgium

**Keywords:** ghrelin, GHSR, stress, anxiety disorders, PTSD, central ghrelin resistance, food intake

## Abstract

Ghrelin is a peptide hormone released by specialized X/A cells in the stomach and activated by acylation. Following its secretion, it binds to ghrelin receptors in the periphery to regulate energy balance, but it also acts on the central nervous system where it induces a potent orexigenic effect. Several types of stressors have been shown to stimulate ghrelin release in rodents, including nutritional stressors like food deprivation, but also physical and psychological stressors such as foot shocks, social defeat, forced immobilization or chronic unpredictable mild stress. The mechanism through which these stressors drive ghrelin release from the stomach lining remains unknown and, to date, the resulting consequences of ghrelin release for stress coping remain poorly understood. Indeed, ghrelin has been proposed to act as a stress hormone that reduces fear, anxiety- and depression-like behaviors in rodents but some studies suggest that ghrelin may - in contrast - promote such behaviors. In this review, we aim to provide a comprehensive overview of the literature on the role of the ghrelin system in stress coping. We discuss whether ghrelin release is more than a byproduct of disrupted energy homeostasis following stress exposure. Furthermore, we explore the notion that ghrelin receptor signaling in the brain may have effects independent of circulating ghrelin and in what way this might influence stress coping in rodents. Finally, we examine how the ghrelin system could be utilized as a therapeutic avenue in stress-related psychiatric disorders (with a focus on anxiety- and trauma-related disorders), for example to develop novel biomarkers for a better diagnosis or new interventions to tackle relapse or treatment resistance in patients.

## The Ghrelin System

### Ghrelin

Ghrelin is a stomach-derived peptide hormone that is tonically released into the blood stream with peak concentrations in response to a negative energy balance (Kojima et al., 1999; Kojima and Kangawa, 2005). The 28-amino-acid hormone is primarily produced by X/A-like oxyntic gland cells in the gastric mucosa from its precursor pre-proghrelin (117 amino acids) (Kojima et al., 1999; Date et al., 2000). In addition, ghrelin expression has also been described in other organs such as the pancreas, kidneys, lungs, heart and placenta (Mori et al., 2000; Gualillo et al., 2001; Gnanapavan et al., 2002; Iglesias et al., 2004; Kageyama et al., 2005).

Ghrelin O-acyltransferase (GOAT) is the enzyme present in the endoplasmatic reticulum of ghrelin-producing cells that ensures the post-translational acylation of desacyl ghrelin (DAG) at the serine-3 residue, resulting in the formation of acyl ghrelin (AG) (Gutierrez et al., 2008). Both, DAG and AG, are released into the blood stream where AG is rapidly converted to DAG (De Vriese et al., 2004), resulting in an AG/DAG ratio of 0.1–0.4 in plasma compared to 2.5 in stomach tissue (Hassouna et al., 2014). While DAG has received less attention in literature, an increasing amount of evidence indicates that it is not only a byproduct of AG synthesis but may act as a separate hormone that modulates or even opposes the effects of AG (for review see Delhanty et al., 2014). For example, DAG was shown to impair the feeding response to peripherally injected AG (Fernandez et al., 2016) and its actions on anxiety and other stress-related behaviors may also be distinct from those of AG (Stark et al., 2016; Mahbod et al., 2018). In this review, we aim to analyze the effects of AG and DAG separately and use the term ‘ghrelin’ whenever it was not possible to make a clear distinction or acylation status was not specified by the authors.

### Ghrelin Receptors (GHSR)

The N-terminus of AG binds with high affinity to the growth hormone secretagogue receptor (GHSR) (Willesen et al., 1999; Cowley et al., 2003), of which two different transcripts have been identified: GHSR-1a and GHSR-1b (McKee et al., 1997). The GHSR-1a, also known as ghrelin receptor (and hereafter simply referred to as ‘GHSR’), is a Gq-protein coupled receptor comprising seven transmembrane α-helical domains to which constitutive activity has been ascribed (Holst et al., 2003). The GHSR-1b form, a truncated splice variant, contains five transmembrane domains and while its physiological role remains poorly understood, it may negatively influence GHSR function by hindering conformational changes (Leung et al., 2007). AG is the main known high-affinity agonist for the GHSR, while (in physiological concentrations) DAG does not appear to act on the GHSR (Bednarek et al., 2000). A recent study indicates that the octanoyl chain is essential for the pharmacological action of AG and that DAG, while it might bind to the GHSR with very low affinity, is unlikely to stabilize an active GHSR conformation (Ferre et al., 2019). Several binding studies propose the existence of a separate receptor specific for DAG in different tissues and also the brain (e.g., Lear et al., 2010; Togliatto et al., 2010; Fernandez et al., 2016). However, such a receptor has not yet been identified (for review see Callaghan and Furness, 2014).

The GHSR is expressed in several organs such as the brain, the anterior pituitary, adrenal and thyroid glands, pancreas and the heart (Guan et al., 1997; Gnanapavan et al., 2002). Within the brain, GHSR expression has been described in several areas including the arcuate nucleus (ARC) and other subregions of the hypothalamus, the ventral tegmental area (VTA), the nucleus tractus solitarius (NTS), the hippocampus and the amygdala (Zigman et al., 2006; Mani et al., 2014). The function the GHSR has in those brain areas and whether they are accessible for circulating ghrelin is not yet fully understood (Perello et al., 2018). Thus, it is essential to note that the GHSR is constitutively active, signaling at 50% of its maximal activity in the absence of its ligand (Holst et al., 2003; Petersen et al., 2009). It is very likely that the function of the ghrelin/GHSR system is not solely dependent on the binding of AG to its receptor, but that also changes in GHSR expression levels might have pervasive biological effects. Furthermore, there is evidence that the GHSR can form heterodimers with various other receptors like for example those for dopamine, serotonin or oxytocin (Schellekens et al., 2013b; Kern et al., 2015; Wallace Fitzsimons et al., 2019), which affects downstream signaling and receptor trafficking (for review see Schellekens et al., 2013a; Abizaid and Hougland, 2020). Recent findings also suggest that the liver-expressed antimicrobial peptide 2 (LEAP2) acts as an endogenous antagonist/inverse agonist of the GHSR that modulates ghrelin function in response to the feeding status (Ge et al., 2018).

### The Ghrelin System and Energy Homeostasis

**FIGURE 1:**
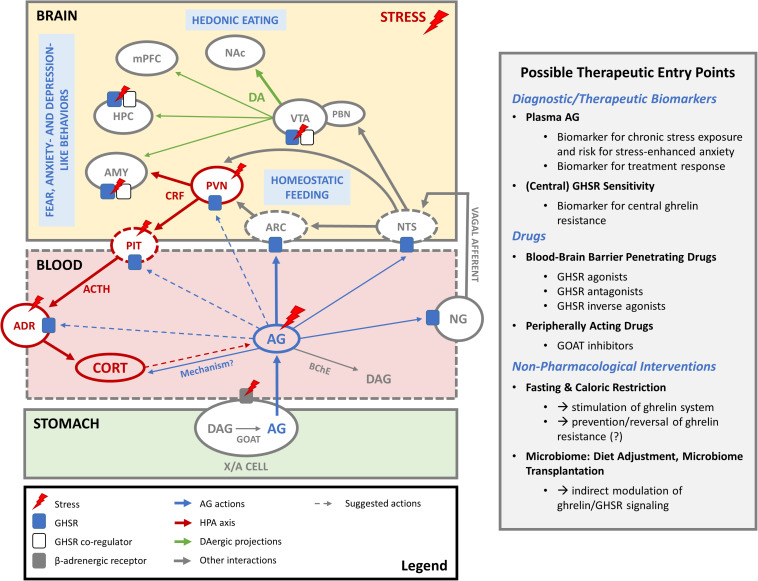
The ghrelin system in stress coping. In periods of hunger and stress, AG is produced by acylation of DAG by GOAT in gastric X/A cells and released into the blood stream. Before it is degraded to DAG, it acts on GHSR in the arcuate nucleus of the hypothalamus (ARC) to regulate homeostatic feeding. Increased AG levels during stress also seem to amplify HPA axis activation and induce corticosterone (CORT) release. The exact mechanism is not clear and could involve indirect activation of CRF-producing neurons in the PVN by inhibition of the local GABAergic tone, modulation of ACTH production in the pituitary (PIT) or direct effects on the adrenal cortex (ADR). AG may also indirectly influence the HPA axis via GHSR in the nodose ganglion (NG) of the vagal nerve or in the nucleus tractus solitarius (NTS). Conversely, whether HPA axis activity during stress has an impact on ghrelin release remains a subject of debate. Elevated AG levels following stress may also be secondary to activation of the sympathetic nervous system and beta receptors on ghrelin-producing X/A cells. Whether AG can pass the blood-brain barrier to act on stress-relevant brain areas where GHSR are expressed is not clear. However, constitutive activity of central GHSR may be regulated via changes in GHSR expression levels or formation of heterodimers with other receptors (e.g., dopamine, serotonin) as co-regulators. Such mechanisms might play a role in stress-related changes in feeding and fear, anxiety- and depression like behaviors, possibly via GHSR in the ventral tegmental area (VTA), hippocampus (HPC) and amygdala (AMY). Also mesocorticolimbic dopaminergic projections (DA) of the VTA to nucleus accumbens (NAc), medial prefrontal cortex (mPFC), HPC and AMY may play a role here. *Abbreviations:* AG, acyl ghrelin; DAG, desacyl ghrelin; GOAT, ghrelin O-acyltransferase; BChE, butyryl cholinesterase; ARC, arcuate nucleus of the hypothalamus; PVN, paraventricular nucleus of the hypothalamus; CRF, corticotropin releasing factor; PIT, pituitary gland; ACTH, adrenocorticotropic hormone; CORT, corticosterone/cortisol; ADR, adrenal cortex; NTS, nucleus tractus solitarius; NG, nodose ganglion; VTA, ventral tegmental area; PBN, parabrachial nucleus; DA, dopamine; NAc, nucleus accumbens; mPFC, medial prefrontal cortex; HPC, hippocampus; AMY, amygdala.

Ghrelin is most widely known for its orexigenic effects. Following its release into the bloodstream, AG can diffuse passively through fenestrated capillaries of the median eminence into the ARC (Schaeffer et al., 2013) where it binds to GHSR that are expressed abundantly on neuropeptide Y/agouti-related peptide (NPY/AGRP) neurons (Figure 1) (Willesen et al., 1999; Cowley et al., 2003). Activation of these hypothalamic neurons by AG potently increases food intake and regulates homeostatic feeding behavior in order to maintain energy balance (Kamegai et al., 2000; Nakazato et al., 2001). Indeed, re-expression of GHSR in AGRP neurons of GHSR KO mice restores, albeit incompletely, the effect of systemic administration of AG on food intake (Wang et al., 2014). In addition, ghrelin also plays an important role in reward-based, hedonic eating behaviors (as reviewed in Perello and Zigman, 2012; Al Massadi et al., 2019), which are very likely mediated by GHSR on dopaminergic neurons of the VTA (Cornejo et al., 2020).

In humans, plasma ghrelin levels are typically increased under low-calorie conditions before meals and fall back to baseline levels after food consumption (Cummings, 2006). Also in rodents, ghrelin plasma levels rise during fasting periods and follow a diurnal rhythm peaking shortly after onset of the dark period, which is the major feeding time for rodents (Murakami et al., 2002; Bodosi et al., 2004). Peripherally circulating ghrelin has various effects on the gastrointestinal tract: it modulates intestinal motility, gastric emptying and gastric acid secretion as well as insulin release from the pancreas (for review see Kojima and Kangawa, 2005).

Overall, the role of ghrelin in driving food intake and energy homeostasis seems well established: it communicates with the brain and the gastrointestinal tract to promote feeding and ensure efficient energy storage and metabolism, lowering the risk of hypoglycemia (reviewed comprehensively by Yanagi et al., 2018). Importantly, while ghrelin signaling stimulates food intake, the effects of a loss of ghrelin signaling on feeding behavior are more subtle if detectable at all (as reviewed in Uchida et al., 2013). Nevertheless, ghrelin appears critical in the regulation of systemic glucose homeostasis and ensures survival after prolonged starvation (Goldstein et al., 2011; McFarlane et al., 2014), potentially through GHSR signaling in the nodose ganglion (NG) of the vagal nerve and the NTS (Scott et al., 2012; Okada et al., 2018) (Figure 1). Projections of the NTS to the parabrachial nucleus (Roman et al., 2016) and to the ARC (Aklan et al., 2020) were shown to regulate food intake in anorectic or hypoglycemic conditions, but it remains poorly understood how the ghrelin system modulates activity in these neurochemically distinct pathways. The effect of ghrelin on glucose homeostasis also involves the release of growth hormone in response to low blood glucose if body fat stores are depleted, which in turn increases glucose release from the liver and contributes to stabilization of blood glucose levels (Goldstein et al., 2011). Moreover, ghrelin modulates pancreatic insulin secretion and regulates its peripheral actions, although the exact mechanisms involved are still a matter of debate (as reviewed by Gray et al., 2019). Interestingly, it has been proposed that a decline in ghrelin system activity may play a role in age-dependent impairment of energy homeostasis and associated disease patterns like reduced appetite, obesity, diabetes or hepatic steatosis, but also cardiovascular dysfunction and neurodegenerative diseases (for review see Stoyanova, 2014; Yin and Zhang, 2016; Amitani et al., 2017).

### Ghrelin/GHSR Interactions With the HPA Axis

Beyond its well-established role as a mediator of feeding behavior, the ghrelin system has also been implicated in many other functions. Notably, it has been proposed as a regulator of stress responses, as its function seems to be closely entwined with that of the hypothalamic-pituitary-adrenal (HPA) axis (for review see Spencer et al., 2015), as illustrated in Figure 1.

Several studies have demonstrated that systemic or central administration of ghrelin increases the plasma concentration of corticosterone in rodents or cortisol in humans (Asakawa et al., 2001a; Stevanovic et al., 2007; Lambert et al., 2011; Cabral et al., 2012, 2016; Jensen et al., 2016). The effects of ghrelin on plasma adrenocorticotropic hormone (ACTH), however, appear inconsistent. One study reported elevated ACTH and an increased volume of ACTH-producing cells in the pituitary following central ghrelin administration (Stevanovic et al., 2007), but another study found no influence of ghrelin on plasma ACTH levels (Jensen et al., 2016). In line with the notion that the ghrelin system may increase activity of the HPA axis, a blunted release of ACTH and corticosterone after exposure to a brief period of immobilization stress was previously observed in ghrelin KO mice (Spencer et al., 2012). Similarly, one study showed lower corticosterone levels in GHSR KO compared to wild type mice in a chronic social defeat stress (CSDS) paradigm (Chuang et al., 2011). In contrast to the aforementioned studies, a more recent publication reported higher corticosterone release after acute restraint stress in non-handled ghrelin KO mice versus wild type controls (Mahbod et al., 2018). Moreover, more elevated plasma corticosterone levels have also been observed in GHSR KO mice following acute and chronic stress paradigms (Patterson et al., 2013a; Mahbod et al., 2018). Interestingly, administration of DAG increased plasma corticosterone levels in non-stressful conditions in ghrelin KO mice, but attenuated corticosterone release during acute stress exposure (Stark et al., 2016), suggesting that the effects of DAG on HPA axis activity may depend on prior stress exposure.

Taken together, the influence of the ghrelin system on HPA axis activity is not fully resolved yet and the underlying mechanisms remain poorly understood. Cabral and colleagues demonstrated that peripheral AG administration induces c-Fos expression not only in the ARC but also in corticotropin releasing factor (CRF)-producing cells in the paraventricular nucleus (PVN) of the hypothalamus (Cabral et al., 2012). These cells, however, do not express GHSR, so in a further study the authors suggested that AG may indirectly activate CRF neurons in the PVN via inhibition of the local GABAergic tone (Cabral et al., 2012, 2016), which was confirmed by a more recent electrophysiological study (Dos-Santos et al., 2018). Ghrelin may also influence the HPA axis through other mechanisms (Figure 1). Intravenous administration of ghrelin appears to inhibit vagal afferents (Asakawa et al., 2001b; Date et al., 2006) and stimulate vagal efferents (Fujitsuka et al., 2011). The influence of the ghrelin system on activity of the vagal nerve remains controversial (Arnold et al., 2006) and may involve activation of GHSR in the NG of the vagal nerve or central mechanisms with activation of GHSR in the NTS (Bansal et al., 2012; Swartz et al., 2014; Su et al., 2019). Further elucidation of the interaction of the ghrelin system with the vagus nerve may be relevant in this context, given that vagal nerve stimulation influences HPA axis activity in rodents (De Herdt et al., 2009) and humans (Warren et al., 2019). Moreover, ghrelin may also have an impact on glucocorticoid production through direct effects on the adrenal gland (Rucinski et al., 2009).

Conversely, how activity in the HPA axis may influence the ghrelin system also remains subject of debate (Figure 1). Administration of an ACTH analog was shown to increase plasma ghrelin levels in humans by increasing cortisol levels (Azzam et al., 2017). On the other hand, chronic immobilization stress increased plasma AG levels in adrenalectomized rats (Meyer et al., 2014), indicating that stress can drive AG release independently of glucocorticoid production. Interestingly, a recent study demonstrated that elevated AG levels following chronic stress exposure could be reversed by the beta receptor antagonist atenolol (Gupta et al., 2019). This hydrophilic beta receptor antagonist does not readily cross the blood-brain barrier, suggesting that stress-induced elevations in plasma AG may be secondary to activation of the sympathetic nervous system.

The notion that ghrelin is released upon stress exposure and mediates at least some behavioral adaptations to stress, which we review and discuss in the following two sections, supports the idea of ghrelin as a novel stress hormone, acting in concert with but also partially independent of the central HPA axis.

## The Role of the Ghrelin System in Stress-Related Behaviors in Rodents

### Effects of Stress on Plasma Ghrelin Levels in Rodents

There is ample evidence showing that plasma ghrelin levels are affected by various acute and chronic stressors, as summarized in Table 1. Increased total ghrelin levels were reported following acute water avoidance stress in rats (Kristenssson et al., 2006, Kristenssson et al., 2007) or maternal separation in mice (Schmidt et al., 2006), while elevated DAG but not AG was observed in plasma of rats following a short immobilization stress episode (Nahata et al., 2014). A number of publications also showed no changes in AG or total ghrelin levels after a single exposure to foot shocks (Harmatz et al., 2016; Rostamkhani et al., 2016) or immobilization stress in rats (Gul et al., 2017). Importantly, nutritional status may also play a role, given that acute stressors such as novelty stress (Saegusa et al., 2011) or a single injection of lipopolysaccharide (LPS) (Stengel et al., 2010) led to a decrease in plasma AG alone or both, DAG and AG, when blood samples were collected in food-deprived conditions.

**TABLE 1:** Effect of stressors on ghrelin levels in rodents.

References	Animals	Feeding status	Duration	Stress paradigm	Ghrelin levels	Time point
(Kristenssson et al., 2006) (Kristenssson et al., 2007)	**rats (WKY, SPD)** f	*ad libitum* fed	**acute**	**water avoidance** 1 h	TG ↑ **(WKY** > **SPD)**	–

(Schmidt et al., 2006)	**mice (CD1)** m/f, pups (p8)	not specified	**acute**	**maternal separation** 4 h, 8 h, 12 h	?G ↑	–

(Nahata et al., 2014)	**mice (ICR)** m	fasted + fed (16 h + 20 min)	**acute**	**immobilization stress** 1 h	AG ↔ DAG ↑	@ during immobilization
		
		fasted (16 h)	**acute**	**immobilization stress** 1 h	AG ↔ DAG ↑	@ during immobilization

(Harmatz et al., 2016)	**rats (LE)** m	*ad libitum* fed	**acute**	**foot shock** 3 × 2s	AG ↔	–

(Rostamkhani et al., 2016)	**rats (WST)** m	*ad libitum* fed	**acute**	**foot shock** 60 × 10s	TG ↔	–

(Gul et al., 2017)	**rats (WST)** m	*ad libitum* fed	**acute**	**immobilization stress** 2 h	?G ↔	–

(Saegusa et al., 2011)	**mice (C57BL/6J)** m	fasted (24 h)	**acute**	**novelty (single housing)** 3 h	AG ↓	–

(Stengel et al., 2010)	**rats (SPD)** m	fasted (17 h)	**acute**	**LPS injection** 100 μg/kg	AG ↓ \ ↔ DAG ↓\↔	@ 2 h, 5 h, 7 h \ 24 h

(Ochi et al., 2008)	**rats (WST)** m	*ad libitum* fed	**subchronic**	**water cage** 5 d	TG ↔ \ ↔ AG ↔ \ ↑ DAG ↔ \ ↔	@ 4 h, 8h, 24 h \ 3 d, 5 d

(Yamada et al., 2015)	**mice (C57BL/6)** m/f	fasted (18 h)	**subchronic**	**social isolation** 7 d	AG ↑ **m** vs. ↔ **f**	–

(Izadi et al., 2018)	**rats (WST)** m	fasted (16–18 h)	**subchronic**	**social stress** 7 d	?G ↔	–
			
			**subchronic**	**social isolation** 7 d	?G ↓	–

(Yam et al., 2017)	**mice (C57BL/6J)** m/f, pups (p2-p14)	not specified	**subchronic**	**early chronic stress** 7 d (p2-p9)	TG ↔ \ ↔ **m** vs. ↑ \ ↓ **f** AG ↔ \ ↔ **m** vs. ↔ \ ↔ **f** DAG ↔ \ ↔ **m** vs. ↑ \ ↓ **f**	@ p9 \ p14

(Lutter et al., 2008)	**mice (C57BL/6J)** m	*ad libitum* fed	**chronic**	**CSDS** 10 d	AG ↑ \ ↑	@ 11d \ 39d

(Chuang et al., 2011)	**mice (GHSR WT/KO)** m, C57BL/6J bckg	*ad libitum* fed	**chronic**	**CSDS** 10 d	AG ↑ (**GHSR KO** and **WT**) DAG ↔ (**GHSR KO** and **WT**)	

(Patterson et al., 2013a)	**mice (C57BL/6J)** m	*ad libitum* fed (STD + HFD)	**chronic**	**CSDS** 21 d	AG ↑ \ ↔	@ 22d \ 62d
	**mice (GHSR WT/KO)** m, C57BL/6J bckg	*ad libitum* fed (STD + HFD)	**chronic**	**CSDS** 21 d	AG ↑ (**GHSR WT** only)	–

(Guo et al., 2019)	**mice (GHSR KO/WT)** m	*ad libitum* fed	**chronic**	**CSDS** 10 d	TG ↑ (**GHSR KO** and **WT**)	–

(Han et al., 2019)	**C57BL/6J mice** m	*ad libitum* fed	**chronic**	**CSDS** 10 d	AG ↑	–
(Razzoli et al., 2015)	**mice (CD1)** m	*ad libitum* fed	**chronic**	**CSDS** 28 d	TG ↓	–

(Lopez Lopez et al., 2018)	**rats (WST)** m	*ad libitum* fed	**chronic**	**CUMS** 20 d, 40 d, 60 d	TG ↔ \ ↑ \ ↔	@ 20 d \ 40 d \ 60 d

(Patterson et al., 2010)	**mice (GHSR WT/KO)** m, C57BL/6J-DBA bckg	*ad libitum* fed (STD + HFD)	**chronic**	**CUMS** 14 d	AG ↑ (**GHSR KO** and **WT**)	–

(Huang et al., 2017)	**mice (C57BL/6J)** m	*ad libitum* fed	**chronic**	**CUMS** 8 w	AG ↑	–

(Elbassuoni, 2014)	**rats (SPD)** m/f	fasted (10h)	**chronic**	**immobilization stress** 20 min/d, 21 d	?G ↑ (**f** > **m**)	–

(Meyer et al., 2014)	**rats (LE)** m	*ad libitum* fed	**chronic**	**immobilization stress** 4 h/d, 14 d	AG ↑	–

(Rostamkhani et al., 2016)	**rats (WST)** m	*ad libitum* fed	**chronic**	**foot shock** 60 × 10s, 15 d	TG ↓	–

(Yousufzai et al., 2018)	**rats (LE)** m	*ad libitum* fed	**chronic**	**immobilization stress** 4 h/d, 14 d	AG ↑	@ 130 d

*Measurements after stressor cessation unless stated otherwise (@ x h/d; 1 = start of stressor). Abbreviations: WKY, Wistar-Kyoto; SPD, Sprague-Dawley; LE, Long-Evans; WST, Wistar; p, postnatal day; bckg, background; STD, standard diet; HFD, high-fat diet; LPS, lipopolysaccharide; CSDS, chronic social defeat stress; CUMS, chronic mild unpredictable stress; TG, total ghrelin; AG, acyl ghrelin; DAG, desacyl ghrelin; ?G, ghrelin (not specified).*

In subchronic stress paradigms like exposure of rats to a flooded cage, elevated AG levels were found (Ochi et al., 2008). Also socially isolated male but not female mice showed increased plasma AG in fasted conditions (Yamada et al., 2015). However, no changes or even decreases in serum ghrelin levels were reported following subchronic social stress or social isolation in rats in a fasted state (Izadi et al., 2018). The effects of stress exposure on plasma ghrelin levels may be dependent on developmental stage and sex, given that subchronic stress induced by limited nesting material seemed to affect plasma ghrelin levels differently in male and female mice, with minor alterations in male pups versus pronounced changes in serum DAG in female pups (Yam et al., 2017).

Regarding chronic stressors, a number of studies reported increased plasma AG levels following CSDS in mice (Lutter et al., 2008; Chuang et al., 2011; Patterson et al., 2013a; Guo et al., 2019; Han et al., 2019). Only one study in mice found decreased total ghrelin levels following CSDS (Razzoli et al., 2015), however, the decrease in total plasma ghrelin was not observed when the food intake was restricted throughout the CSDS paradigm. Similarly, chronic mild unpredictable stress (CUMS) protocols were shown to induce elevated total serum ghrelin levels in rats (Lopez Lopez et al., 2018) and plasma AG levels in mice (Patterson et al., 2010; Huang et al., 2017). Also chronic immobilization stress increased ghrelin or AG levels in rats (Elbassuoni, 2014; Meyer et al., 2014). A recent study showed that elevated plasma AG levels following chronic immobilization stress in adolescent rats persisted up to four months after stress exposure (Yousufzai et al., 2018). Only one study reported decreased total ghrelin levels following repeated exposure to foot shocks (Rostamkhani et al., 2016). In summary, while literature on the effects of acute or subchronic stress exposure on plasma ghrelin levels is conflicting, chronic exposure to stress relatively consistently increases plasma AG levels.

### Effects of Stress on Food Intake and Body Weight in Rodents and the Possible Role of Ghrelin and GHSR Signaling

Exposure to acute and chronic stressors impacts on food intake and body weight in rodents and the ghrelin system has been proposed a role in stress-related feeding behaviors, which, however, is not fully established yet. In Table 2 we give an extensive, although certainly not exhaustive, overview of the effects of different acute and chronic stress paradigms on food intake and body weight.

**TABLE 2:** Effect of stressors on food intake in rodents.

References	Animals	Paradigm *(Feeding status)*	Food intake (FI)	Time point *Measurement*	Body weight (BW)	Time point *Measurement*
**Acute stress paradigms**						

(Meerlo et al., 1996)	**rats (TMDS3)** m	**social defeat** 1x or 2x	↓ (**2x** only)	@ 1 d-3 d *FI daily*	↔ \ ↓ (**2x** > **1x**)	@ 1 d \ 2 d-3 d *BW g*

(Gul et al., 2017)	**rats (WST)** m	**immobilization stress** 2 h *(fed)*	↓	@ 24 h *FI cumul*	↓	@ 24h *BW change %*

(Saegusa et al., 2011)	**mice (C57BL/6J)** m	**novelty** 3 h (*fasted, 24h)*	↓ \ ↔	@ 1 h, 3 h \ 6 h *FI cumul*	-	-

4*(Matsumoto et al., 2017)	**mice (C57BL/6)** f, aged	**novelty** 3 h *(fasted, 18 h)*	↓	@ 1 h, 3 h *FI cumul*	-	-
	
	**mice (C57BL/6)** m/f, aged	**novelty** 3 h *(fed)*	↓ **m** vs. ↔ **f**	@ 6 h, 24 h *FI cumul*	-	-

(Stengel et al., 2010)	**rats (SPD)** m	**LPS injection** 100 μg/kg (*fasted, 16h)*	↓	@ 2 h, 5 h, 7 h, 24 h *FI per time period*	-	-

**(Sub)chronic stress paradigms**						

(Jeong et al., 2013)	**mice (ICR)** m	**immobilization stress** 2 h/d, 15 d	↓ \ (↓)^*ns*^	@ 2 d-6 d \ 7 d-15 d *FI daily*	↔	@ 2 d-15 d *BW g*

(Jiang and Eiden, 2016)	**mice (PACAP WT)** m, C57BL/6N bckg	**immobilization stress** 1 h/d, 7 d	↓	@ 7 d *FI cumul*	↓	@ 7 d *BW loss %*

(Li et al., 2015)	**mice (ICR)** f, maternal	**immobilization stress** ? h/d, 21 d	(↓)^*ns*^	@ 1 d-16 d *FI daily*	↓	@ 3 d-16 d *BW g*

(Mograbi et al., 2020)	**rats (WST)** m	**immobilization stress** 2,4 or 6 h/d, 21 d	-	-	↓	@ 1 w, 2 w, 3 w *BW gain %*

(Ochi et al., 2008)	**rats (WST)** m	**water cage** 5 d	-	-	↓	@ d1-d5 *BW change %*

4*(Izadi et al., 2018)	**rats (WST)** m	**social stress** 7 d	↔	@ 8 d *FI cumul (3 h)*	↔	@ 7 d *BW change g*
	
	**rats (WST)** m	**social isolation** 7 d	↓	@ 8 d *FI cumul (3 h)*	↓	@ 7 d *BW change g*

(Rostamkhani et al., 2016)	**rats (WST)** m	**foot shock** 60 × 10 s, 15 d	↔ \ ↓ \ (↓)^*ns*^	@ 2 d \ 7 d \ 15 d *FI cumul (24 h)*	↔ \ ↓	@ d1 \ d15 *BW g*

(Yam et al., 2017)	**mice (C57BL/6J)** m/f, pups (p2-p14)	**early chronic stress** 7 d (p2-p9)	-	-	↓ \ ↑	@ p2-p9 \ p9-p14 *BW gain g*

(Elbassuoni, 2014)	**rats (SPD)** m/f	**immobilization stress** 20 min/d, 21 d	↑ (**m** > **f**)	@ 1 d-21 d *FI daily avgd*	↑ (**m** > **f**)	@ 21d *BW gain g*
(Patterson et al., 2010)	**mice (GHSR WT/KO)** m, C57BL/6J-DBA bckg	**CUMS** 14 d *(STD* + *HFD)*	↓ ↔ **GHSR-KO**	@ 1 d-14 d *kcal daily avgd*	↓ ↔ **GHSR-KO**	@ d14 *BW change g*

(Yao et al., 2016)	**mice (C57BL/6N)** m	**CUMS + social isolation** 21 d *(fasted, 12 h)*	↓	@ 21 d *FI cumul (24 h)/BW*	-	-

(He et al., 2018)	**rats (SPD)** f	**CUMS** 4 w	-	-	↓	@ 1 w, 2 w, 3 w, 4 w *BW change %*

(Yan et al., 2018)	**mice (C57BL/6J)** m	**CUMS** 21 d	↔ \ ↓	@ 7 d, 14 d \ 21 d *FI cumul (24 h) ?*	↔ \ ↓	@ 7 d, 14 d \ 21 d *BW g*

(Forbes et al., 1996)	**rats (LH)** m	**CUMS** 6 w	-	-	↓	@ 1 w-6 w *BW g*

(Harris et al., 1997)	**rats (WST)** m	**CUMS** 40 d	↔	@ 1 d-40 d *FI daily*	↔ \ ↓	@ 1 d-3 d \ 4 d-40 d *BW g*

(Lopez Lopez et al., 2018)	**rats (WST)** m	**CUMS** 60 d	↔	@ 1 d-60 d *FI daily*	↔ \ ↓	@ 1 d-40 d \ 40 d-60 d *BW g*

8*(Simas et al., 2018)	**rats (WST)** m	**CUMS** 4 w *(STD)*	↔	@ 1 w, 2 w, 3 w, 4 w *FI daily avgd*	↔ (but no BW gain from 0w as control)	@ 1 w, 2 w, 3 w, 4 w *BW g*
	
		**CUMS** 4 w *(HFD)*	↔ \ ↓ \ (↓)^*ns*^	@ 1 w, 2 w \ 3 w \ 4 w *FI daily avgd*	↔ \ ↓	@ 1 w, 2 w, 3 w \ 4 w *BW g*

(Li et al., 2019)	**rats (WST)** m	**CUMS** 4 w	-	-	↔ \ ↓ (and ↓ BW gain from 1 w)	@ 1 w \ 2 w, 3 w, 4 w *BW g*

(Yun et al., 2020)	**mice (C57BL/6N)** m	**CUMS** 6 w	-	-	↔ \ ↓	@ 2 w \ 4 w, 6 w *BW change g*

(Huang et al., 2017)	**mice (C57BL/6J)** m	**CUMS** 8 w	↑	@ 0 w-8 w *(1x/w) FI daily avgd*	↔	@ 8 w *BW change g*

(Lutter et al., 2008)	**mice (GHSR WT/KO)** m, C57BL/6 J bckg	**CSDS** 10 d	↑ ↔ **GHSR KO**	@ 1 d-10 d, 11 d-13 d *FI daily avgd*	↔	@ 1 d-13 d *BW change g*

5*(Patterson et al., 2013a)	**mice (C57BL/6J)** m	**CSDS** 21 d *(STD* + *HFD 4h/d)*	↑ **STD** vs. ↓ **HFD**	@ 1 d-21 d *FI daily avgd*	*↑*	@ 1 d-21 d *BW g avgd*
	
	**mice (GHSR WT/KO)** m, C57BL/6J bckg	**CSDS** 21 d *(STD* + *HFD 4h/d)*	↑ ↔ **GHSR KO**	@ 1 d-21 d *kcal daily avgd*	*↑* ↔ **GHSR KO**	@ 21 d *BW gain g*

(Patterson et al., 2013b)	**mice (C57BL/6J)** m	**CSDS** 21 d	↑	@ 1 d-21 d *FI and kcal daily avgd*	↔	@ 21 d *BW, BW change g*

5*(Razzoli et al., 2015)	**mice (C57BL/6J)** m	**CSDS** 4 w	↑	@ 1 w-4 w ? *FI daily avgd*	-	-
	**mice (CD1)** m	**CSDS** 4 w *(STD 1 w*, *HFD 3 w)*	↑ \ ↔	@ 1 w, 2 w, 3 w \ 4 w *kcal daily avgd ?*	↔ \ ↑	@ 1 w \ 2 w, 3 w, 4 w *BW g*
(Chuang et al., 2011)	**mice (GHSR WT/KO)** m, C57BL/6J bckg	**CSDS** 10 d	**STD** ↔ vs. **HFD** ↑↔ **GHSR KO**	@ 26 d (during CPP) *FI cumul (CPP)*	*↑* ↔ **GHSR KO**	@ 26 d (during CPP) *BW change % (CPP)*

(Berton et al., 1998)	**rats (LEW)** m	**RI SDS** 7 d	↓ \ (↓)^*ns*^	@ 1 d-5 d \ 6 d-7 d *FI daily (g/100g)*	↓ \ ↔	@ 1 d-4 d \ 5 d-6 d *BW change %*

(Fanous et al., 2011)	**rats (SPD)** m	**RI SDS** every 3 d, 10 d	-	-	↓	@ 10 d *BW gain g*

(Patki et al., 2013)	**rats (SPD)** m	**RI SDS** 7 d	↔	@ 1 d-7 d *FI daily avgd*	↓	@ 7 d *BW gain g*

*Acute paradigms: Measurements after stressor cessation (@ x h/d/w; 1 = start of stressor). Chronic paradigms: Measurements during paradigm and/or after stressor cessation as indicated (@ x h/d). Abbreviations: TMDS3, Tryon Maze Dull S3; WST, Wistar; SPD, Sprague-Dawley; bckg, background; p, postnatal day; LH, Lister hooded; LEW, Lewis; LPS, lipopolysaccharide; CUMS, chronic mild unpredictable stress; STD, standard diet; HFD, high-fat diet; CSDS, chronic social defeat stress; RI, resident-intruder; SDS, social defeat stress; FI, food intake; cumul, cumulative; avgd, averaged; BW, body weight; ()^*ns*^, non-significant.*

Acute stressors such as a single session of social defeat (Meerlo et al., 1996), immobilization stress (Gul et al., 2017), novelty stress (Saegusa et al., 2011; Matsumoto et al., 2017) but also LPS injection (Stengel et al., 2010) were mostly found to reduce food intake. While only a few of these studies measured body weight, they reported a decrease following exposure to an acute stressor (Meerlo et al., 1996; Gul et al., 2017).

Also in subchronic and chronic stress paradigms food intake and body weight are typically negatively affected. For example, repeated immobilization stress decreased food intake (Jeong et al., 2013; Jiang and Eiden, 2016) and in parallel decreased (Li et al., 2015; Jiang and Eiden, 2016; Mograbi et al., 2020) or had no effect on body weight (Jeong et al., 2013). Also exposure to a flooded cage for five days decreased body weight (Ochi et al., 2008) and, similarly, social isolation for a week (Izadi et al., 2018) or chronic administration of foot shocks (Rostamkhani et al., 2016) reduced food intake and body weight. Early life stress induced by limited access to bedding and nesting material for a week led to attenuated weight gain during stress exposure in male and female pups, which was compensated by an increased weight gain after termination of the stress exposure (Yam et al., 2017). As an exception, only one study found increased food intake and body weight gain during immobilization stress over a period of three weeks, which was more pronounced in male than female rats (Elbassuoni, 2014).

Exposure to CUMS, which utilizes a battery of different stressors over a time course of several weeks, relatively consistently led to decreases in food intake (Patterson et al., 2010; Yao et al., 2016; He et al., 2018; Yan et al., 2018) and body weight in mice and rats (Forbes et al., 1996; Harris et al., 1997; Patterson et al., 2010; Lopez Lopez et al., 2018; Simas et al., 2018; Yan et al., 2018; Li et al., 2019; Yun et al., 2020). A few studies reported decreases in body weight or body weight gain, while food intake was not significantly altered (Harris et al., 1997; Lopez Lopez et al., 2018; Simas et al., 2018). Only one study found an increase in daily chow consumption and no differences in body weight between CUMS and non-CUMS mice after eight weeks of stress exposure (Huang et al., 2017). Interestingly, Simas et al. reported a decreased food intake in mice exposed to CUMS compared to non-stressed mice when only a high-fat diet (HFD) was provided, but not when fed a standard diet (Simas et al., 2018). This suggests that the rewarding properties of calorically dense food are abolished during CUMS. In line with this notion, several of the previously mentioned studies also evaluated sucrose or saccharine preference. Some found no differences (Forbes et al., 1996; Harris et al., 1997; Yun et al., 2020), but in several studies CUMS exposure led to reduced sucrose or saccharine intake (Yao et al., 2016; Huang et al., 2017; He et al., 2018; Yan et al., 2018), which may reflect stress-induced anhedonia.

On the other hand, CSDS in mice quite robustly led to increases in food intake (Lutter et al., 2008; Patterson et al., 2013a, b; Razzoli et al., 2015), associated with an increase in body weight in some studies (Patterson et al., 2013a; Razzoli et al., 2015) but not in others (Lutter et al., 2008; Patterson et al., 2013b). Chuang and colleagues showed that conditioned place preference (CPP) for HFD was significantly increased in CSDS-exposed mice, which was associated with a higher intake of HFD chow and a greater weight gain over the course of the CPP paradigm (Chuang et al., 2011). However, in contrast to these studies in mice, subchronic and chronic social defeat paradigms in rats resulted in decreases in daily food intake (Berton et al., 1998) and body weight (Berton et al., 1998; Fanous et al., 2011; Patki et al., 2013).

Some of the aforementioned studies also investigated the role of the GHSR in stress-related changes in feeding behavior and found that both increases (CSDS) or decreases (CUMS) in food intake and body weight gain were abolished in GHSR KO mice (Lutter et al., 2008; Patterson et al., 2010, 2013a). Moreover, increased CPP for and higher intake of HFD and an associated weight gain after CSDS exposure were not present in GHSR KO mice (Chuang et al., 2011), indicating a central role of ghrelin signaling in these stress-induced changes. The increased CPP, however, could be reinstated by selective re-expression of GHSR in tyrosine hydroxylase-positive neurons, which the authors concluded are very likely involved in mediating stress-induced food reward. Interestingly, a recent study used re-expression of GHSR in dopamine transporter (DAT)-positive neurons of GHSR KO mice to confirm that GHSR signaling in dopaminergic neurons controls appetitive and consummatory behaviors toward HFD in *ad libitum* fed mice (Cornejo et al., 2020). Indeed, it has been proposed previously that the ghrelin system may play an important role in stress-induced food reward behavior and act as a critical modulator at the interface of homeostatic control of appetite and food reward during stress, promoting hyperphagia and hedonic intake of calorically dense ‘comfort foods’ (Schellekens et al., 2012).

Even though stress paradigms typically increase AG levels and in some instances increase feeding, paradoxically, exposure to stress may also be associated with a decrease in food intake and body weight. During acute stress exposure, it is likely that the orexigenic effects of high plasma AG are overcome by other factors, such as stress-induced changes in CRF signaling in the brain (Jochman et al., 2005; Haque et al., 2013; Dunn et al., 2015; Mogami et al., 2016). Chronic stress, on the other hand, might induce central ghrelin resistance, associated with lower GHSR expression in particular brain areas (Harmatz et al., 2016), which then may further contribute to reduced food intake and anhedonia-like behaviors in a GHSR-dependent manner, but irrespective of high circulating AG concentrations. This may apply for physical stressors like foot shocks, immobilization stress or various stressors used during CUMS which are typically associated with reduced food intake and decreases in body weight. The same hypothesis, however, does not seem to hold true for CSDS in mice, where high plasma AG is mostly associated with increases in food intake and where GHSR signaling in dopaminergic neurons has been proposed to mediate stress-induced food reward as a coping mechanism (Chuang et al., 2011). The observation that CSDS increases food intake and/or body weight specifically in mice but not in rats could be attributed to species differences in social dominance behavior (Kondrakiewicz et al., 2019). The question remains what impact circulating ghrelin and central GHSR signaling may have on other stress-related behaviors in rodents.

### Role of Circulating Ghrelin in Fear, Anxiety- and Depression-Like Behaviors

While some studies propose that ghrelin reduces fear, anxiety- and depression-like behaviors in rodents, others actually suggest an opposite role. One early study showed that central administration of ghrelin antisense DNA had anxiolytic and antidepressant-like effects and reduced the retrieval of conditioned fear in unstressed rats (Kanehisa et al., 2006). Similarly, Spencer and colleagues found reduced anxiety-like behaviors in ghrelin KO mice under baseline conditions, but increased anxiety scores following acute immobilization stress (Spencer et al., 2012). However, a more recent study also found higher anxiety-like behavior in ghrelin KO mice under non-stressed conditions (Mahbod et al., 2018). A genetic deletion of GOAT and thus attenuated AG levels (Zhao et al., 2010) reduced anxiety-like behavior prior to stress exposure (Mahbod et al., 2018) or was anxiogenic regardless of stress conditions (Stark et al., 2016).

Pharmacological studies investigating the effects of AG and other GHSR ligands have also delivered contradictory results. Systemic or central injection of a single dose of AG has been shown to induce anxiety- and depression-like behaviors in mice and rats that were not previously subjected to stress (Asakawa et al., 2001a; Carlini et al., 2002, 2004; Currie et al., 2012, 2014; Brockway et al., 2016; Jackson et al., 2019). Similarly, central infusion of ghrelin for one month increased anxiety- and depression-like behavior in rats (Hansson et al., 2011). In contrast, a few other studies reported anxiolytic and antidepressant-like effects of a single systemic dose of AG in non-stressed mice (Lutter et al., 2008; Jensen et al., 2016). In a recent publication, the administration of AG into the dorsal raphe nucleus was anxiolytic in an inhibitory avoidance test but facilitated learned escape behavior in rats (Cavalcante et al., 2019).

In studies where AG is administered systemically, it should generally be considered that it can be converted to DAG (De Vriese et al., 2004). This could be relevant, given that DAG was found to have anxiogenic effects in ghrelin KO mice which were not previously subjected to stress (Stark et al., 2016). Moreover, the effects of exogenous AG administration may also depend on feeding state. An injection of ghrelin into the amygdala had no significant effect in fed mice, but was anxiolytic when no food was available (Alvarez-Crespo et al., 2012), suggesting that AG may promote foraging and risk-taking in response to a negative energy balance.

When focusing on the effects of exogenously administered ghrelin following stress exposure, an anxiolytic effect appears more evident. Peripherally administered AG attenuated anxiety-like behavior and stress responses after exposure to air puffs or immobilization stress (Jensen et al., 2016). Interestingly, DAG also produced anxiolytic-like effects following stress exposure in ghrelin KO mice (Stark et al., 2016). Moreover, anxiety- and depression-like behaviors induced by CUMS were at least partly rescued by administration of ghrelin or the ghrelin agonist GHRP-6 (Huang et al., 2017, 2019). A recent study demonstrated that intra-hippocampal administration of ghrelin fully and intraperitoneal injection partly normalized stress-enhanced anxiety- and depression-like phenotypes in a CSDS paradigm (Han et al., 2019). However, another study found that reduced social interaction following CSDS exposure could not be rescued by subcutaneous administration of AG or ghrelin agonist GHRP-2 (Gupta et al., 2019).

A number of studies also investigated how GHSR ligands affect fear conditioning and extinction. Administration of the GHSR agonist MK0677, systemically or directly into the basolateral amygdala, around the time of fear conditioning reduced contextual and cued fear memory strength in unstressed rats, while the GHSR antagonist D-Lys3 increased it (Harmatz et al., 2016). An earlier study of the same group, however, showed that repeated systemic or intra-amygdalar injection of MK0677 led to an enhancement of cued fear memory strength (Meyer et al., 2014). In rats that were subjected to chronic immobilization stress, stress-induced increases in cued fear were abolished when the GHSR antagonist D-Lys3-GHRP-6 was administered daily before stress exposure (Meyer et al., 2014). In their 2016 study, this group reported that increased cued fear in chronically stress-exposed rats was associated with reduced binding of fluorescently labeled ghrelin in the basolateral amygdala, which they defined as central ghrelin resistance (Harmatz et al., 2016). Therefore, the authors hypothesized that a stress-induced elevation of AG levels and prolonged GHSR activation may lead to impaired GHSR signaling in the brain via downregulation of GHSR expression (Harmatz et al., 2016). Interestingly, infusion of MK0677 into the lateral amygdala was also shown to improve cued fear extinction in non-stressed mice (Huang et al., 2016). However, in our recently published study, we did not find significant effects of systemic or intra-VTA administration of MK0677 on cued fear expression or extinction in non-stressed mice (Pierre et al., 2020). Importantly, as the GHSR is constitutively active in the absence of ghrelin (Holst et al., 2003; Petersen et al., 2009), it is therefore important to consider the effects of central GHSR signaling independently of circulating AG or exogenous administration of an GHSR agonist.

### Role of GHSR Signaling in Fear, Anxiety- and Depression-Like Behaviors

Under non-stressed conditions, no or only marginal effects on fear, anxiety- and depression like behaviors were observed in GHSR KO mice compared to wild type controls (Mahbod et al., 2018; Guo et al., 2019; Lu et al., 2019; Pierre et al., 2019). Nevertheless, one study found anxiolytic and antidepressant-like effects following overexpression of GHSR in the basolateral amygdala in non-stressed mice (Jensen et al., 2016). Another study reported reduced fear memory retention after contextual fear conditioning in unstressed GHSR KO mice (Albarran-Zeckler et al., 2012), but this observation was not confirmed in a more recent study (Hornsby et al., 2016).

Clearer evidence for behavioral effects in mice lacking the GHSR was obtained following stress exposure, where most studies reported impaired stress coping. For example, mild stressors like air puffs induced an exacerbated avoidance response in GHSR KO mice (Jensen et al., 2016). Similarly, CSDS resulted in more pronounced depression-like behavior in GHSR KO mice, as assessed by a social avoidance test (Lutter et al., 2008; Chuang et al., 2011). A local knockdown of the GHSR in the hippocampus also aggravated anxiety- and depression-like behaviors in mice following exposure to a CSDS or CUMS paradigm (Han et al., 2019; Huang et al., 2019), pointing to a protective role of the GHSR during chronic stress.

### Effects of Caloric Restriction on Fear, Anxiety- and Depression-Like Behaviors

Caloric restriction and food deprivation paradigms typically increase plasma ghrelin levels and were also used in several studies to manipulate the endogenous ghrelin system and thus indirectly study its effects on fear, anxiety- and depression-related behaviors in rodents. An initial study demonstrated anxiolytic and antidepressant-like effects of caloric restriction that were abolished in GHSR KO mice (Lutter et al., 2008). In a later publication, the same group showed that the neuroprotective agent P7C3 restored the antidepressant-like effect of caloric restriction in GHSR KO mice, potentially by increasing hippocampal neurogenesis (Walker et al., 2015). This suggests that the effects of caloric restriction on depression-like behaviors may depend on GHSR signaling in the hippocampus and ensuing neurogenesis. Recently, a study reported that acute food deprivation for 24 h and chronic caloric restriction had anxiolytic- and antidepressant-like effects in wild type mice, which were abolished after re-feeding (Lu et al., 2019). In GHSR-KO mice, however, acute food deprivation enhanced, while only chronic caloric restriction reduced anxiety- and depression-like responses. This led the authors to conclude that the positive effect of acute but not chronic fasting was GHSR-dependent (Lu et al., 2019). The finding that the anxiolytic-like action of 24 h food deprivation in wild type mice was neutralized by administration of a GHSR antagonist further supported this conclusion (Lu et al., 2019).

In fear conditioning paradigms, caloric restriction and overnight fasting before extinction training were shown to enhance fear extinction and suppress return of fear in mice (Riddle et al., 2013; Huang et al., 2016; Verma et al., 2016), an effect that was abolished by administration of the GHSR antagonist D-Lys3 into the lateral amygdala (Huang et al., 2016). Interestingly, also in humans spontaneous recovery and reinstatement of fear were reduced after an overnight fast, which was correlated with ghrelin plasma levels (Shi et al., 2018). However, we could not confirm the extinction-promoting effect of overnight fasting using a weak extinction protocol and also found no difference between wild type and GHSR KO mice (Pierre et al., 2020). Thus, while manipulation of GHSR signaling may be an interesting avenue to curb fear expression or promote fear extinction, it appears that the experimental conditions under which such effects can be observed remain uncertain.

### Summary, Shortcomings and Future Directions of Rodent Studies Investigating the Role of the Ghrelin System in Stress-Related Behaviors

The often mixed findings of rodent studies make it challenging to draw a general conclusion about the role of ghrelin and GHSR in stress-related behaviors. Possible reasons for these inconsistencies are diverse: the use of different mouse or rat strains, different age or sex of the animals, varying breeding, handling or habituation conditions, different time of day for testing and use of diverse behavioral and stress paradigms. The feeding status of the animals during testing may be of particular importance, as administration of exogenous ghrelin or GHSR agonists may confound the readout of behavioral assays by the instatement of hunger.

In summary, published literature suggests that the ghrelin system plays a different role in individuals depending on the amount and quality of previous stress exposure. This seems most obvious for GHSR signaling, which does not appear to affect fear, anxiety- and depression-like behaviors under non-stressed conditions but may exert mostly protective effects during chronic stress. A similar trend can be observed in pharmacological studies using GSHR ligands, where, without prior stress exposure, GHSR agonists predominantly promote anxiety- and depression-like behaviors, but seem to reduce chronic stress-related phenotypes. Also, caloric restriction may have beneficial effects on fear, anxiety- and depression-like behaviors in unstressed rodents through activation of GHSR. Nevertheless, the response of rodents to the potentially stressful nature of food deprivation might additionally challenge stress coping mechanisms and, together with differences in fasting procedures, contribute to inconsistencies in literature.

It has been proposed that prolonged elevation of plasma AG levels during chronic stress induces a central ghrelin resistance with reduced GHSR expression (Harmatz et al., 2016), which would impede ligand-dependent as well as ligand-independent GHSR signaling and thereby eliminate its protective role during stress. In the face of central ghrelin resistance, a rescue of stress-related phenotypes by AG or GHSR agonists as well as caloric restriction, which elevates endogenous ghrelin levels, seems unlikely. Nevertheless, many studies suggest beneficial effects of GHSR agonism and fasting on fear, anxiety-, and depression-like behaviors.

Only a few studies have considered the role of DAG in stress coping, although it constitutes the majority of total circulating plasma ghrelin and may have behavioral effects that are distinct from those of AG. This applies not only for feeding behaviors (Fernandez et al., 2016) but also for stress-related behaviors (Stark et al., 2016). This may be relevant during chronic stress and associated central ghrelin resistance, as AG can be rapidly converted to DAG (De Vriese et al., 2004), which appears to not act via the GHSR (Bednarek et al., 2000; Ferre et al., 2019). Thus, the reported beneficial effects of exogenous AG administration during chronic stress paradigms in rodents may be attributed to DAG rather than AG action. In this context, it also can be noted that the way of reporting plasma ghrelin levels has not been consistent in existing literature. Studies often report either only total or AG levels or do not specify acylation status at all, thereby hindering the overview of ghrelin alterations in baseline versus stressed conditions.

One more point to consider is that the GHSR may have functions independent from binding of AG, which is of particular importance since accessibility of many GHSR-expressing brain areas for the ghrelin peptide is not resolved yet (Perello et al., 2018). While the endogenous peptide LEAP2 has been shown to act as an antagonist/inverse agonist of the GHSR (Ge et al., 2018), it remains unknown to what extent it influences GHSR activity in different brain areas. Because of its high constitutive activity (Holst et al., 2003; Petersen et al., 2009) also changes in GHSR expression levels will accordingly alter activation of downstream signaling pathways as well as a possible formation of heterodimers with other receptors, such as receptors for dopamine and serotonin (Schellekens et al., 2013b; Kern et al., 2015). Given that heterodimer formation influences receptor signaling and trafficking (for review see Schellekens et al., 2013a; Abizaid and Hougland, 2020), this could serve to fine-tune GHSR signaling in response to certain neurotransmitters and, conversely, GHSR signaling would also affect these neurotransmitter systems. These complex (inter)actions of the GHSR could further result in individual differences, where an impairment of GHSR signaling occurs in stress-susceptible but not stress-resilient individuals, in which GHSR agonism still would have beneficial effects. Therefore, it might be useful to identify such individual differences in GHSR sensitivity and investigate which factors contribute to constitutive GHSR signaling.

Furthermore, this requires a clear distinction between findings from studies manipulating peptide hormone levels or using GHSR ligands (e.g., ghrelin KO mice, GOAT KO mice, pharmacological studies) versus studies conducted in GHSR KO mice. For a comprehensive dissection of the role of GHSR signaling in distinct neuronal populations, the development of new biomolecular tools like GHSR-Cre mice is indispensable in order to perform targeted pharmaco- and optogenetic manipulations and selectively employ new viral techniques. Such novel genetic and viral tools could also be used to create conditional knockouts of GHSR, GOAT or ghrelin. It cannot be excluded that in conventional germ-line knockouts compensatory mechanisms are at play that disguise the loss-of-function effect, thereby confounding results and leading to mixed findings and wrong conclusions.

## The Ghrelin System as a Novel Therapeutic Avenue in Stress-Related Psychiatric Disorders?

### A Need for New Treatment Strategies: Ghrelin as a Vantage Point?

In this review, we aimed to provide an overview of the current literature on the role of ghrelin system in stress coping. We summarized findings on fear, anxiety- and depression-like behaviors in rodent models and in this final chapter, we want to discuss the possible therapeutic utility of the ghrelin system in stress-related psychiatric disorders, with a focus on anxiety disorders and post-traumatic stress disorder (PTSD). They are the most common cause of psychiatric illness, with an estimated lifetime prevalence of up to 30% (Kessler et al., 2005a, 2012) and women being affected almost twice as often as men (Baxter et al., 2013). Besides reducing the quality of life of patients and their families dramatically, they cause an immense socioeconomic burden in modern western societies (Wittchen et al., 2011; Olesen et al., 2012). However, a lack of major advances in psychopharmacotherapy over the past three decades as well as limited validity of current symptom-based diagnostic manuals (Lang et al., 2016) leave anxiety disorders and PTSD poorly diagnosed and undertreated (Craske et al., 2017). Moreover, a large number of anxiety patients do not show long-term benefit from currently available treatments and fail to achieve full remission or often relapse with time (Hofmann and Smits, 2008; Bandelow et al., 2015; Koek et al., 2016; Craske et al., 2017). Currently, there are also no biomarkers for the measurement of treatment response available (Craske et al., 2017). Thus, there is an urgent need for the development of novel strategies to augment existing diagnostic and treatment options to improve their efficiency and tackle under- and misdiagnosis as well as treatment resistance (Singewald et al., 2015; Sartori and Singewald, 2019).

There is a growing interest in the involvement and therapeutic potential of the ghrelin system in psychiatric diseases. Positioned at the interface between feeding circuitry, metabolism and the HPA axis, its dysregulation could not only potentially influence food intake and energy homeostasis but also stress vulnerability. This involvement of the ghrelin system in different physiological processes has sparked a discussion about its role in a variety of pathological conditions like obesity and eating disorders, but also addictive disorders as well as anxiety disorders, PTSD and mood disorders (for review see Labarthe et al., 2014; Wittekind and Kluge, 2015; Bali and Jaggi, 2016; Stievenard et al., 2016; Jiao et al., 2017; Shi et al., 2017). The potential role of the ghrelin system is further underscored by the notion that mood-, anxiety- and trauma-related disorders are often associated with metabolic dysregulation and also show a high level of comorbidity with obesity or eating disorders (Scott et al., 2008; Ng et al., 2013; Mason et al., 2014; Meng and D’Arcy, 2015; Aaseth et al., 2019). It is very likely that comorbid diseases share a common neurobiological pathology, which could be of use to gain a better understanding of underlying disease mechanisms and to identify potential therapeutic targets.

### (Dysregulation of) the Ghrelin System in Stress-Related Psychiatric Disorders

In humans, acute exposure to stressors is associated with elevated plasma ghrelin concentrations. In healthy individuals but also in obese patients with binge-eating disorder, total plasma ghrelin levels were elevated in those individuals who also had a high cortisol response to a Trier social stress test (TSST) (Rouach et al., 2007). Similarly, two studies with only female participants found elevated plasma cortisol and AG levels in healthy women (Raspopow et al., 2010) and higher saliva ghrelin but not cortisol levels in women suffering from bulimia nervosa (Monteleone et al., 2012) after stress exposure in a TSST. Other studies with only male participants reported no changes or, after alcohol consumption, even decreased AG levels following a TSST (Zimmermann et al., 2007) and no changes in total plasma ghrelin levels before and after a forced arithmetic task in a cohort of lean and obese men (Lambert et al., 2011). Taken together, these studies strongly suggest that the effects of acute stress exposure on plasma ghrelin levels may be dependent on sex. While the effects of chronic stress exposure have not been investigated explicitly in humans, one translational study reported elevated AG in teenagers 4.5 years after experiencing a severe traumatic event and proposed high plasma AG levels as a long-lasting biomarker for chronic stress exposure and a perpetuating risk factor for stress-enhanced fear (Yousufzai et al., 2018).

In line with the notion that alterations of the ghrelin system may be a biomarker for stress exposure, several other studies have investigated possible dysregulations of the ghrelin system in stress-related psychiatric disorders such as anxiety disorders, PTSD or major depressive disorder (MDD). The Gln90Le polymorphism in the pre-proghrelin gene has been linked to an increased risk of panic disorder (Hansson et al., 2013), while, in contrast, the Leu72Met variant of the pre-proghrelin gene was not associated with panic disorder (Nakashima et al., 2008) but with symptom severity in PTSD (Li et al., 2018) and MDD (Nakashima et al., 2008). A recent study reported elevated plasma ghrelin levels in children with a diagnosis of an anxiety disorder, which was more pronounced in girls than boys (Ozmen et al., 2019). Interestingly, ghrelin levels, serum cortisol and anxiety scores together with body mass index were more reduced in obese children following a combined dietary and mindfulness intervention in comparison to a control group that only underwent a dietary intervention (Lopez-Alarcon et al., 2020). However, in female patients diagnosed with anorexia nervosa or obesity, ghrelin levels were not correlated with depression- and anxiety-like symptoms independently of weight and body fat (Lawson et al., 2012). Numerous studies investigated plasma ghrelin levels in patients with MDD, which is often comorbid with anxiety- and trauma-related disorders and associated with stress-induced changes in food intake (Kessler, 1997, Kessler et al., 2005b; Kendler et al., 1999; Grant et al., 2013). They found increased fasting AG (Kurt et al., 2007; Algul and Ozcelik, 2018) and total ghrelin (Ozsoy et al., 2014), but also decreased fasting AG and DAG (Barim et al., 2009) or no changes in fasting AG or total ghrelin levels at all (Schanze et al., 2008; Kluge et al., 2010; Gimenez-Palop et al., 2012; Matsuo et al., 2012). Two of these studies showed a positive correlation of eating behavior with total ghrelin levels in MDD patients (Schanze et al., 2008; Matsuo et al., 2012), while another one reported lower AG levels in depressed patients with increased appetite (Simmons et al., 2020), indicating that plasma AG may not be good predictor for appetite or food intake in depressed patients.

The effect of treatment on ghrelin plasma levels in stress-related psychiatric disorders (mainly MDD) has also been examined. One study that found reduced plasma AG and DAG when comparing untreated MDD patients with healthy controls reported no effect of citalopram on ghrelin levels (Barim et al., 2009). Maprotiline treatment in male lean MDD patients, on the other hand, resulted in mildly increased plasma ghrelin levels (Pinar et al., 2008). In a mixed patient group suffering from bipolar disorder or MDD, serum AG levels were elevated compared to controls and decreased after electroconvulsive therapy (Kurt et al., 2007). A similar decrease in total ghrelin levels was reported in another study where patients received electroconvulsive therapy and/or different antidepressant drugs (Ozsoy et al., 2014). Interestingly, one study found, that therapy resistance in MDD and panic disorder was correlated with elevated AG (Ishitobi et al., 2012) and the effect of lithium augmentation treatment in treatment-resistant MDD could be characterized by a slight decrease in plasma AG in responders compared to an increase in non-responders (Ricken et al., 2017).

The conflicting findings in these studies probably result from confounding factors like nutritional status and time of day at sample collection, gender, age, body mass index, comorbid disorders and drug treatments, such as the type of antidepressant. Moreover, not all studies distinguished between AG and DAG and only measured total ghrelin levels or did not specify acylation status. Overall, it remains inconclusive whether genetic variants in the ghrelin system are causally involved in stress-related psychiatric disorders and whether altered ghrelin plasma levels are a predisposing factor for or just a byproduct of these diseases. Elevated plasma AG could indicate prior chronic stress exposure (Yousufzai et al., 2018), which in turn may enhance the risk for mental health disorders. Nevertheless, plasma AG appears to correlate with treatment resistance, suggesting more than mere association. Moreover, experiments in rodents indicate that stress-induced activation of the ghrelin system not only influences feeding but may also have a direct impact on fear, anxiety- and depression-like behaviors.

### Therapeutic Potential of Targeting the Ghrelin System in Stress-Related Psychiatric Disorders

Despite the role of the ghrelin system in stress-related psychiatric disorders is not fully resolved yet, it provides a new and interesting vantage point in the search for more efficient diagnostic and therapeutic strategies (also see Figure 1). It remains unknown whether a modulation of the ghrelin system could be beneficial in the treatment of stress-related psychiatric disorders, however multiple pharmacological tools to do so have become available over the last two decades (McGovern et al., 2016; Lee et al., 2020; Moose et al., 2020). This includes GHSR agonists and antagonists as well as inverse agonists and GOAT inhibitors, although no authorized medicine is commercially available to date. The pharmacological characterization of many ligands has only just begun and it will be of great importance to consider their central penetrance and possible signaling bias and further elucidate downstream signaling in order to allow targeted predictive modeling and the design of future ghrelin-based therapies (Ramirez et al., 2019; Howick et al., 2020). To our knowledge, there are currently no studies published that directly investigated the effect of ghrelin-targeted interventions in stress-related psychiatric conditions. One study in healthy human participants found enhanced extinction memory retention and reduced return of fear following an overnight fast, which was negatively correlated with ghrelin plasma levels (Shi et al., 2018). Whether fasting may improve exposure-based psychotherapy outcomes in patients with anxiety disorders or PTSD and whether this would be associated with changes in plasma AG concentrations or brain GHSR function remains to be established.

As a biomarker, high plasma AG could indicate prior stress exposure (Yousufzai et al., 2018), which in turn may enhance the risk for stress-related psychiatric disorders. While plasma AG concentrations were not consistently associated with disease, they may predict treatment resistance (Ishitobi et al., 2012; Ricken et al., 2017). In several studies, a decrease in plasma AG or total ghrelin levels during therapy was also associated with treatment response (Kurt et al., 2007; Ozsoy et al., 2014; Ricken et al., 2017; Lopez-Alarcon et al., 2020). A lack of changes in plasma AG concentrations may thus reflect decreased sensitivity of the GHSR and dysregulation of its feedback system. In light of our previous discussion that GHSR in the brain may act partly independent of circulating AG, this highlights the therapeutic potential for the development of diagnostic tools to study brain GHSR function in order to aid further stratification of patients for an optimization of treatment strategies.

It also should be kept in mind that potentially beneficial effects associated with activation of the ghrelin system, as observed in some rodent experiments, may be lost in patients with impaired GHSR function (Harmatz et al., 2016). To this end, a precise definition of the phenomenon of central ghrelin resistance and corresponding neurobiological correlates is indispensable. Present literature merely suggests an impaired feeding response following ghrelin (agonist) administration, associated with reduced c-Fos expression in the ARC (Briggs et al., 2010) and a reduction of AG binding (Harmatz et al., 2016) as markers for disrupted GHSR function. In addition, it is presently unclear whether fasting or other interventions could rapidly reverse central ghrelin resistance. A loss of GHSR function in hypothalamic NPY/AgRP neurons as a result of diet-induced obesity could be rescued by caloric restriction (Briggs et al., 2010, 2013), but so far no study investigated whether this can be extrapolated to stress-induced central ghrelin resistance in brain regions beyond the hypothalamus. Instead of treatment, also a prevention of central ghrelin resistance could be considered. For example, a study in rats showed that administration of a GHSR antagonist during a chronic immobilization paradigm prevented the emergence of stress-enhanced fear (Meyer et al., 2014), while the same paradigm without GHSR antagonist administration led to reduced GHSR expression in the basolateral amygdala, which hampered the attenuation of stress-enhanced fear memories by a GHSR agonist (Harmatz et al., 2016). Therefore, preservation of GHSR function during chronic stress may keep the ghrelin system targetable for treatment interventions.

Also indirect targeting of the ghrelin system by other approaches may be of relevance in stress-related psychiatric disorders, for example via the gut microbiome (Schalla and Stengel, 2020). Changes in diet and gut microbiota composition have been linked to changes in plasma ghrelin levels in rodents and humans (for review see Lach et al., 2018). Moreover, it has been proposed that short-chain fatty acids produced by gastrointestinal microbiota may compete with octanoate for binding at the GOAT and interfere with the acylation of ghrelin (Heiman and Greenway, 2016). A recent study showed that different microbiota-derived metabolites, amongst them also short-chain fatty acids, may also directly attenuate GHSR signaling (Torres-Fuentes et al., 2019). Interestingly, one clinical study demonstrated that the use of dietary capsaicin altered gut microbiota composition and reduced ghrelin levels in healthy subjects (Kang et al., 2016). This is of particular interest, given that elevated plasma AG may reflect a higher risk for the development of stress-related psychiatric conditions (Yousufzai et al., 2018). Overall, this highlights the possibility of utilizing the gut microbiome to influence ghrelin signaling and thereby modulate the gut-brain axis in stress, although the underlying mechanisms how gut microbiota interact with the ghrelin system are not fully resolved yet.

We believe that the therapeutic potential of ghrelin-targeted interventions in stress-related psychiatric disorders is just starting to be explored. Especially a better understanding of the interactions of ghrelin with the HPA axis, the role of DAG versus AG and factors that control constitutive GHSR activity or lead to ghrelin resistance in the brain may uncover novel diagnostic and therapeutic avenues that have not been considered so far.

### Interaction of Ghrelin With Neurotransmitter Systems as a Gateway to the Treatment of Psychiatric Disorders

Abundant evidence demonstrates interaction of the ghrelin system with different neurotransmitters, neuromodulators and their receptors, including monoamines, neuropeptides and endocannabinoids (Brunetti et al., 2002; Skibicka et al., 2012b; Edwards and Abizaid, 2016; Hedegaard and Holst, 2020). One possibility how the ghrelin system may affect behaviors relevant for psychiatric disorders is through its effect on the limbic system. These effects may result from direct activation of GHSR in different limbic areas like hippocampus or amygdala but may also be the consequence of activation of GHSR in neurotransmitter systems projecting to these limbic areas. In this regard, the effect of ghrelin signaling on the release of noradrenalin, serotonin and dopamine merits further discussion. Only few studies have investigated the effect of ghrelin on noradrenalin and serotonin release. One study found that peripheral ghrelin may activate the noradrenergic pathway from the hindbrain to the hypothalamus (Date et al., 2006) and another study reported that food deprivation and central administration of ghrelin augmented the increase of noradrenalin release in the PVN following foot shock stress in rats (Kawakami et al., 2008). Concerning ghrelin’s effects on serotonin signaling, one study showed that ghrelin postsynaptically depolarized serotonin neurons in rat brain slices containing the dorsal raphe nucleus (Ogaya et al., 2011), while a second study found that ghrelin reduced the firing rate of dorsal raphe neurons in brain slices prepared from rats chronically treated with ghrelin (Hansson et al., 2011). Interestingly, acute central administration of ghrelin in mice was shown to increase serotonin turnover and the expression of serotonin-related genes in the amygdala (Hansson et al., 2014). While these studies demonstrate that ghrelin influences serotonin and noradrenaline signaling, the direct implications for fear, anxiety- and mood-related behaviors remain unclear.

Because the interaction of the ghrelin with the dopamine system is studied more extensively, we will further highlight certain aspects of this interaction and, as an example, discuss possible therapeutic relevance. Ghrelin was shown to modulate the spontaneous firing frequency of mesolimbic dopaminergic neurons in the VTA (Abizaid et al., 2006) and trigger dopamine release in the nucleus accumbens (NAc), predominantly in the shell region (Jerlhag et al., 2007; Jerlhag, 2008; Quarta et al., 2009; Cone et al., 2014). Associated with this rise of extracellular dopamine levels in the NAc following systemic or intra-VTA administration of ghrelin, stimulation of locomotor activity and conditioned place preference was observed (Jerlhag et al., 2007; Jerlhag, 2008). GHSR antagonism or a ghrelin KO, on the other hand, attenuated alcohol-, cocaine-, amphetamine- or morphine-induced locomotor stimulation and CPP and abolished the associated increase of accumbal dopamine release (Jerlhag et al., 2010; Jerlhag et al., 2011; Engel et al., 2015). In paradigms offering a free choice between standard chow and rewarding (palatable) food, ghrelin in the VTA stimulated hedonic feeding, but not homeostatic food intake (Egecioglu et al., 2010; Skibicka et al., 2011, 2013; Schele et al., 2016). The mechanism via which ghrelin entrains the midbrain dopaminergic system, however, is not fully resolved yet. Recently, it was shown that ghrelin-binding cells are present in most subnuclei of the VTA, but not in the NAc (Cornejo et al., 2018). Moreover, Cornejo and colleagues demonstrated that centrally but not peripherally injected ghrelin recruits specific subsets of dopaminergic neurons in the parabrachial nucleus and GABAergic neurons in the interfascicular nucleus of the VTA (Cornejo et al., 2018). As for many other brain areas where the GHSR is expressed, there is no clear evidence whether peripherally circulating ghrelin reaches the VTA and directly acts on midbrain dopaminergic neurons (Perello et al., 2018). Also indirect mechanisms of activation (e.g., via orexigenic neurons in the lateral hypothalamus) have yet to be elucidated (Quarta and Smolders, 2014; Al Massadi et al., 2019). Furthermore, it has been proposed that (hippocampal) dopamine signaling is dependent on heterodimerization between dopamine D1 (DRD1) and GHS receptors, regulating DRD1-induced hippocampal synaptic plasticity and learning (Kern et al., 2015).

The role of ghrelin signaling on mesolimbic dopamine was previously reviewed extensively in the context of food reward (Skibicka and Dickson, 2011; Perello and Zigman, 2012; Skibicka et al., 2012a; Liu and Borgland, 2015; Al Massadi et al., 2019). However, these actions of ghrelin may also be relevant for stress-related psychiatric disorders. A possible role in mood disorders seems likely, given that ghrelin has been proposed to mediate CSDS-induced food reward (Chuang et al., 2011) and protect against depressive-like symptoms following CSDS in mice (Lutter et al., 2008). This is further corroborated by the observation that hyperphagic depressed patients showed a greater change in AG in response to a meal, which was associated with increased activity in the VTA (Cerit et al., 2019). As such, ghrelin signaling on mesolimbic dopamine neurons may indeed constitute the interface of stress, mood and food reward (Schellekens et al., 2012). With respect to anxiety disorders and PTSD, the abundant expression of the GHSR and GHSR mRNA on/in midbrain dopamine neurons of the VTA (Zigman et al., 2006; Mani et al., 2014) is particularly interesting (Figure 1), as accumulating evidence suggests midbrain dopaminergic signaling as an important player in fear extinction (Abraham et al., 2014; Kalisch et al., 2019). The extinction of fear is the driving mechanism behind the reduction of fear responses and very basis of exposure-based cognitive behavioral therapy (Milad and Quirk, 2012), which is first-line in the treatment of anxiety- and trauma-related disorders (Craske, 2010; Craske et al., 2017). Also translationally, this paradigm is very appealing as it is easy to model in the laboratory in rodent models as well as humans (Milad and Quirk, 2012; Singewald and Holmes, 2019). Moreover, impaired fear extinction was suggested as a relevant endophenotype for anxiety disorders and PTSD (Singewald and Holmes, 2019). Recently, it has been proposed that during extinction learning midbrain dopaminergic neurons encode a quasi-appetitive reward prediction error, signaling the omission of the aversive unconditioned stimulus as a ‘better-than-expected’ outcome (Luo et al., 2018; Salinas-Hernandez et al., 2018; Cai et al., 2020). Besides this temporally restricted role in extinction acquisition, dopamine was also suggested to play a key role in the consolidation phase in a human fMRI study (Gerlicher et al., 2018). In a collaborative study, our lab has previously shown that enhancing dopaminergic signaling by L-DOPA treatment helps the formation of long-lasting extinction memories and prevents return of fear in extinction-competent mice and healthy humans (Haaker et al., 2013). Moreover, we were able to show that L-DOPA treatment can rescue deficient fear extinction in a mouse model for refractory anxiety disorders (Whittle et al., 2016).

Despite this clear evidence of interaction of the two systems, very few studies have investigated the possible benefits of targeting the ghrelin-dopamine axis in stress-related psychiatric disorders. We were able to show recently, that administration of the ghrelin agonist MK0677 directly into the VTA induces dopamine release in the NAc but also in amygdala and medial prefrontal cortex, which are important parts of fear and anxiety circuitries (Pierre et al., 2020). But despite increasing dopamine release in VTA projection areas, in our hands, MK0677 did not influence fear acquisition or extinction and extinction memory retrieval in unstressed mice. One explanation could be that the achieved rise in dopamine may not have been sufficient or not timed adequately, which may be enhanced by optimizing ghrelin agonists, dosage and ways of administration. Since the elucidation of the exact role of dopamine during fear extinction has only begun, advances in this field could help to utilize the ghrelin-dopamine axis in a more targeted manner (e.g., timing, individual differences) in future studies.

## Conclusion and Future Directions

Although the body of literature about the ghrelin system is growing rapidly, a great amount of questions regarding its effect on brain function remain unanswered. This complicates the evaluation of pathological relevance and therapeutic utility of ghrelin and GHSR in stress-related psychiatric disorders. To this end, a better understanding of the role of the ghrelin system in stress coping and its interplay with the HPA axis will be important. Further studies will be necessary to characterize brain GHSR signaling beyond the action of AG on its receptor, as DAG and ligand-independent GHSR activity have received little attention in existing literature. Moreover, the phenomenon of central ghrelin resistance in chronic stress needs to be studied more intensely in order to establish associated neurobiological correlates. The notion that ghrelin modulates fear, anxiety- and depression-like behaviors as a stress hormone seems to arise mostly from rodent experiments, while evidence from human studies is sparse and inconclusive. Nevertheless, it appears evident that ghrelin is much more than a ‘hunger hormone’ and represents an interesting target for novel interventions in stress-related psychiatric disorders that will become more accessible once its dynamics in the context of stress are better understood.

## Author Contributions

EMF and DDB conceptualized the review and performed literature research. EMF wrote the manuscript with contributions and supervision from DDB. DDB and NS reviewed and revised all sections. All authors read and approved the final manuscript.

## Conflict of Interest

The authors declare that the research was conducted in the absence of any commercial or financial relationships that could be construed as a potential conflict of interest.

## Abbreviations

ACTH: adrenocorticotropic hormone
AG: acyl ghrelin
AGRP: agouti-related peptide
ARC: arcuate nucleus
CPP: conditioned place preference
CRF: corticotropin-releasing factor
CSDS: chronic social defeat stress
CUMS: chronic mild unpredictable stress
DAG: desacyl ghrelin
GABA: gamma aminobutyric acid
GHSR: ghrelin receptor (growth hormone secretagogue receptor)
GOAT: ghrelin-O-acyltransferase
HFD: high-fat diet
HPA: hypothalamic-pituitary-adrenal
KO: knockout
LEAP2: liver-expressed antimicrobial peptide 2
LPS: lipopolysaccharide
MDD: major depressive disorder
NAc: nucleus accumbens
NG: nodose ganglion
NPY: neuropeptide Y
NTS: nucleus tractus solitarius
PTSD: post-traumatic stress disorder
PVN: paraventricular nucleus
TSST: Trier social stress test
VTA: ventral tegmental area.
